# Mechanosensory and mechanotransductive processes mediated by ion channels in articular chondrocytes: Potential therapeutic targets for osteoarthritis

**DOI:** 10.1080/19336950.2021.1903184

**Published:** 2021-03-29

**Authors:** Kun Zhang, Lifu Wang, Zhongcheng Liu, Bin Geng, Yuanjun Teng, Xuening Liu, Qiong Yi, Dechen Yu, Xiangyi Chen, Dacheng Zhao, Yayi Xia

**Affiliations:** Department of Orthopedics, Orthopaedics Key Laboratory of Gansu Province, Lanzhou University Second Hospital, Lanzhou Gansu, China

**Keywords:** Ion channel, mechanical force, articular chondrocyte, membrane potential, osteoarthritis

## Abstract

Articular cartilage consists of an extracellular matrix including many proteins as well as embedded chondrocytes. Articular cartilage formation and function are influenced by mechanical forces. Hind limb unloading or simulated microgravity causes articular cartilage loss, suggesting the importance of the healthy mechanical environment in articular cartilage homeostasis and implying a significant role of appropriate mechanical stimulation in articular cartilage degeneration. Mechanosensitive ion channels participate in regulating the metabolism of articular chondrocytes, including matrix protein production and extracellular matrix synthesis. Mechanical stimuli, including fluid shear stress, stretch, compression and cell swelling and decreased mechanical conditions (such as simulated microgravity) can alter the membrane potential and regulate the metabolism of articular chondrocytes via transmembrane ion channel-induced ionic fluxes. This process includes Ca^2+^ influx and the resulting mobilization of Ca^2+^ that is due to massive released Ca^2+^ from stores, intracellular cation efflux and extracellular cation influx. This review brings together published information on mechanosensitive ion channels, such as stretch-activated channels (SACs), voltage-gated Ca^2+^ channels (VGCCs), large conductance Ca^2+^-activated K^+^ channels (BK_Ca_ channels), Ca^2+^-activated K^+^ channels (SK_Ca_ channels), voltage-activated H^+^ channels (VAHCs), acid sensing ion channels (ASICs), transient receptor potential (TRP) family channels, and piezo1/2 channels. Data based on epithelial sodium channels (ENaCs), purinergic receptors and N-methyl-d-aspartate (NMDA) receptors are also included. These channels mediate mechanoelectrical physiological processes essential for converting physical force signals into biological signals. The primary channel-mediated effects and signaling pathways regulated by these mechanosensitive ion channels can influence the progression of osteoarthritis during the mechanosensory and mechanoadaptive process of articular chondrocytes.

## Introduction

Articular chondrocytes are subjected to phasic stimulation by mechanical forces that is due to normal walking/mobility in all manners. These biomechanical forces cause articular cartilage deformation. Articular cartilage is a layer of low-friction, load-bearing tissue that functions as a cushion for sensing body weight and exercise. Beneficial mechanical stimuli promote cartilage regeneration and prevent or attenuate osteoarthritis progression. In contrast, harmful mechanical stimuli can disrupt cartilage homeostasis and can accelerate cartilage degeneration. Moreover, chondrocyte senescence is associated with age-related osteoarthritis characterized by impaired cartilage repair [1].

The synovium is a thin sheet of cellular, well-vascularized connective tissue that lines the synovial joint cavity. Fibroblast-like synoviocytes in the synovium synthesize and secrete hyaluronan (HA) into the joint fluid to ensure/promote joint lubrication, which is essential for articular cartilage health. Increased intra-articular osmotic pressure can drastically increase the hydraulic conductance of the stretched synovial lining (increased area, reduced thickness) [2]. The major part of the hydraulic resistance to fluid transport between blood and joint cavity is generated by the synovial lining and interstitial HA, which can conserve intra-articular lubricant and lead to outflow buffering via the osmotic pressure of an HA concentration polarization layer on the synovial surface [3–10]. Static stretch stimulated HA secretion in primary rabbit synoviocyte cultures from microdissected synovial intima [11]. The activation of Ca^2+^ influx-dependent activation of the PKCɑ-MEK-ERK1/2 cascade is importantly involved in mechanically induced HA secretion by static stretch in synoviocytes [12], which suggests that mechanically induced Ca^2+^ influx and Ca^2+^-dependent kinases can regulate synovial HA secretion [13]. Mechanically induced HA secretion by cyclic joint movement may protect joints against the damaging effects of repetitive joint use and replace the HA lost during periods of immobility, which may contribute to the clinical benefit of exercise therapy in moderate osteoarthritis [14]. The epithelial sodium channel (ENaC) channel blocker amiloride inhibited mechanically induced HA secretion in synoviocytes [15]. Ca^2+^-dependent kinases are major regulators of synovial HA secretion and fibroblast-like synoviocytes cultured from the inner synovium of rabbits exhibit voltage-dependent inward and outward currents involving voltage-gated (Kv1.1) K^+^ channels and L-type VGCCs [13].

Cyclic tensile strain upregulates the mRNA levels of cyclooxygenase-2 (COX-2), collagen 2 and aggrecan (ACAN) and induces the release of nitric oxide and prostaglandin E 2 in monolayer cultures of porcine articular chondrocytes. In addition, anabolic modulator transforming growth factor beta3 (TGFβ3), catabolic modulator TGFβ1 and matrix metalloproteinase-1 (MMP-1) all increase over the duration of static mechanical stretch [16]. Physiologically, dynamic compression-induced membrane strain has been shown to have anabolic effects on chondrocytes and to maintain articular cartilage in a healthy state [17]. Ion channel-mediated flux of Ca^2+^ can regulate the proliferation of human chondrocytes [18]. Mechanical forces stimulate the synthesis and release of matrix proteins and glycosaminoglycan in articular cartilage via complex molecular mechanisms, including mechanical regulation of ion channels such as K^+^ channels, Ca^2+^ channels and Na^+^/K^+^ pumps and stretch-activated channels (SACs). The common denominator of this channel activity is often mechanical activation of the Ca^2+^ signaling pathways [19–27]. A lack of mechanosensitive ion channels, such as ENaC and transient receptor potential vanilloid 4 (TRPV4) has been shown to impair the mechanical sensitivity of chondrocytes in response to mechanical membrane strain induced by hypotonic solution [23,28–30]. Mechanical forces such as compression, strain, extracellular matrix (ECM) deformation, substrate deflection and fluid shear stress in the mechanical cartilage environment can significantly alter the expression and activity of membrane mechanosensitive ion channels resulting in intracellular cation mobilization of chondrocytes and regulation of the proliferation and differentiation of immature chondrocytes and survival of hypertrophic chondrocytes [19,31–36]. Some mechanosensitive ion channels sense and transduce mechanical transduction in response to physiological levels of mechanical force. However, abnormal or excessive mechanosensitive ion channel activity may accelerate the deterioration of chondrocytes and ECM in response to injurious levels of mechanical stimulus.

For example, repeated overloading leads to oxidant-dependent mitochondrial dysfunction in chondrocytes, and may result in destabilization of cartilage and osteoarthritis (OA) by disrupting chondrocyte anabolic responses to mechanical stimuli [37]. Mechanical stimulation (such as physiological compression) significantly modulates the expression of collagen 2, ACAN, the SOX9 transcription factor, cartilage oligomeric matrix protein, collagen degradation marker C2C and vascular endothelial growth factor in OA human cartilage [38]. Excessive mechanical loading of subchondral osteoblasts altered the phenotypic characteristics of chondrocytes accompanied by upregulation of MMP genes and downregulation of proteoglycan and collagen in porcine chondrocytes [39]. Appropriate expression of ion channels is vital for the formation of extracellular matrix, for instance, K^+^ channel gene transcription is altered in OA [27,40].

**Figure 1.**
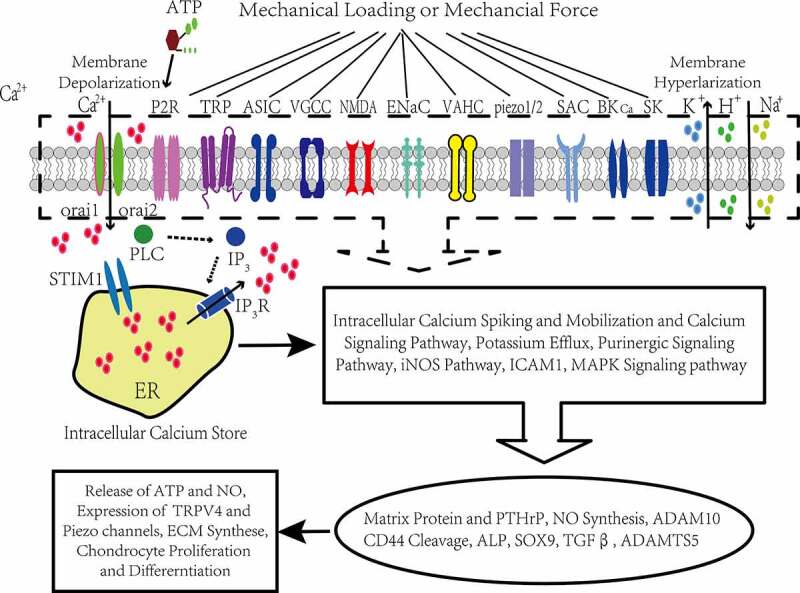
Conceptual illustration of the cellular mechanotransduction mechanism mediated by ion channels in articular chondrocytes

There are differences in electrophysiological properties and gene expression between human chondrocytes and chondroprogenitors derived from normal and osteoarthritic cartilage [41]. Elucidating the mechanosensitive molecular mechanisms by which these mechanical stimuli regulate chondrocyte membrane ion channels and joint homeostasis will provide insights into the pathophysiological process of OA [42–44]. However, the mechanobiology of articular chondrocytes is still not well understood partly because of the complexity of the mechanotransduction process in articular cartilage [43,45]. Adopting shorter step lengths during daily activity and when walking for exercise can reduce mechanical stimuli associated with articular cartilage degenerative processes in adults with and without obesity [46]. Mechanically induced release of growth factor responds quickly to mechanical damage and repairs ligament tissue by activating transcription factor 2 accompanied by accelerated repair of ligament injury repair, promoted ligament fibroblast migration of OA, decreased MMP-2 activity and remitted cell deformation [47]. Mechanically induced release of growth factor may protect articular chondrocytes against harmful cell stress responses and prevent OA progression. Walking with reduced step length may benefit adults at risk for disability because of knee osteoarthritis suggesting that physiologic joint loading helps maintain cartilage integrity. However, both disuse and overuse can result in cartilage degradation. The primary characteristics of OA are destruction of articular cartilage, ECM degradation, dysfunction of chondrocytes, osteophyte formation and subchondral bone alterations [48]. An osteophyte is a fibrocartilage-capped bony outgrowth that is one of the features of OA [49]. Moreover, osteophytes are thought to originate from progenitor cells (residing in the periosteum at the boundary of bone and cartilage) that undergo a process of chondrogenesis and finally endochondral ossification [50]. Osteophytes can be induced with a single mechanical impact applied to the periosteum in rat knees, which indicates that moderate trauma to the periosteal layer of the joint may play a role in osteophyte development [51]. Periarticular osteophyte formation may erode articular cartilage by disrupting chondrocyte functions accelerating ECM degradation in the context of OA progression (Figure 1).

## The mechanosensory processes in chondrocytes

Chondrocytes are the unique cells found in all types of cartilage. Key to their function is the ability to respond to mechanical loads with changes in metabolic activity. This mechanosensory and mechanotransductive process is, in part, mediated through the activity of a range of expressed mechanosensitive transmembrane ion channels.

The following transmembrane ion channels have been reported to be expressed in human, equine, bovine, chicken, and murine chondrocytes: transient receptor potential (TRP) family channels, including TRPV1, TRPV2, TRPV3, TRPV4, TRPV6 and TRPP1/2; large conductance Ca^2+^-activated K^+^ channels (BK_Ca_ channels); small-conductance Ca^2+^-activated K^+^ channels (SK_Ca_); SACs; voltage-activated H^+^ channels (VAHCs); T/L-type voltage-gated Ca^2+^ channels (VGCCs); Piezo1/2 channels; epithelial sodium channels (ENaCs); purinergic receptors; Ca^2+^ release-activated Ca^2+^ channels (CRACs) and chloride channels.

Physiological ion homeostasis is fundamental to routine chondrocyte functions, ion transport through membrane channels is vital for electrophysiological properties manifested by altered membrane potential and mechanically induced Ca^2+^ influx and activation of the Ca^2+^-related signaling pathways in response to mechanical stimulation [52], and compression increases ACAN-mRNA via Ca^2+^/calmodulin-dependent signaling processes in bovine articular chondrocytes (BACs) [53]. K^+^ channel subfamilies, such as BK_Ca_ channels, SK_Ca_ channels and Kv subtype channels, have all been identified in articular chondrocytes and participate in mechanotransduction, cell volume regulation, apoptosis and chondrogenesis [40]. TRPV4 and Piezo1/2 channels mediate mechanical modulation of articular chondrocytes and are involved in hypertrophic chondrocyte and ECM degeneration. Many studies have demonstrated that chondrocytes, as nonexcitable cells, sense and respond to a variety of mechanical forces by different morphological and metabolic changes, and that these forces are essential for the mechanical properties of articular cartilage [35,54–60]. In addition to ion channels, cytoskeletal elements, connexin and pannexin-based hemichannels form mechanosensitive units along with adjacent purinergic receptors, and mediate the mechanotransduction process of chondrocytes involving ATP release and intracellular Ca^2+^ oscillation [21,45,61,62]. ENaCs and SACs participate in the mechanical electrophysiological process of the chondrocyte membrane along with cytoskeletal proteins, ɑ5β1 integrins via mitogen-activated protein kinase (MAPK) signaling, tyrosine kinases and phospholipase C (PLC), which phosphorylate inositol 1,4,5-trisphosphate (IP_3_), focal adhesion kinase (FAK), paxillin (a focal adhesion complex adapter protein) and β-catenin [63–68]. The cyclical pressure-induced strain activated SK_Ca_ channels and SACs involving the phosphorylation of p125, p90, p70, FAK, β-catenin and ɑ5 integrin in normal human articular chondrocytes [56]. ENaCs and VGCCs colocalized with β1 integrins mediate mechanotransduction processes along with the cytoskeleton in murine chondrocytes [69]. Mechanical stimuli activate MAPK signals and alter ACAN gene expression most likely via ion channels [23,70]. The association of mechanosensitive ion channels with integrin, cytoskeletal and signaling complexes is vital for physiological mechanically induced responses of chondrocytes including inducing intracellular anion oscillations and activating mechanical signaling pathways. Maintenance of cell volume is essential for survival and the membrane potential may be vital for regulating chondrocyte volume by changing the osmotic pressure for ionic flux [71]. Mechanically induced cell volume changes induce ATP release in chondrocytes, and purinergic receptors are activated [72], and a Gq-mediated PLC enzymatic reaction is triggered, which produces IP_3_ that binds to IP_3_ receptors and induces Ca^2+^ release from the endoplasmic reticulum [21]. The depletion of intracellular Ca^2+^ stores causes subsequent store-operated Ca^2+^ entry (SOCE) through CRACs consisting of Orai1, Orai2 and stromal interaction molecule 1 (STIM1) and causes membrane hyperpolarization [21,73–76]. CRAC-modified mesenchymal stem cells (MSCs) have distinct differentiation fates to adipocytes, osteoblasts, and chondrocytes from multipotent mesenchymal stem cells [77].

In summary, these channels can sense and respond to environmental factors especially mechanical cues, such as ECM deformation and membrane strain [23,27,35,56,57,78–86]. As shown in Table 1, ion channels that are involved in the mechanosensory and mechanotransductive processes of articular chondrocytes are summarized and the mechanosensory and mechanotransductive processes are briefly described.

**Table 1.** The mechanosensory and mechanotransductive process of articular chondrocytes mediated by ion channels

Study	Cell/Tissue	Ion channel and mechanical stimuli	Mechanosensory and mechanotransductive process and mechanoadaptation
Wu et al (2000)	Growth plate chondrocytes	SACs, L-type VGCCs, stretch-induced cartilage matrix deformation	Synthesis of matrix protein, chondrocyte proliferation and differentiation
Srinivasan et al. (2015)	T-type VGCC KO mice and WT mice, MC3T3-E1	T-type VGCCs, dynamic compression	Matrix protein, mechanically induced OA phenotype
Parisi et al. (2018)	Embryonic chick hindlimb explants of chick articular joint	SACs, L-type VGCCs, dynamic compression	Ca^2+^ influx, mechanically induced cellular responses
Tanaka et al. (2005)	rat growth plate chondrocytes	Ca^2+^ channels sensitive to nifedipine, mechanical strain	Parathyroid hormone-related protein, chondrocyte maturation and matrix formation
Yellowley et al. (2002)	BACs	SACs, membrane stretch	Ca^2+^ influx, altered membrane potential
Chowdhury et al (2006)	BACs	Purinergic 2 receptor ion channels, SACs, dynamic compression	ATP release, Ca^2+^ influx, inhibition of NO-induced catabolic effect on articular cartilage
Millward-Sadler et al (2004)	Normal human chondrocytes	P2Y2 purinergic receptor, SK channels, mechanical stimulation at 0.33 Hz for 20 minutes	ATP release, membrane hyperpolarization
Lee et al. (2000)	Normal human articular chondrocytes	SK channels, SACs, 0.33 Hz cyclical pressure-induced strain	Phosphorylation of p125, p90, and p70, FAK, β-catenin and integrin
Mouw et al. (2007)	BACs	K^+^ channels, Ca^2+^ channels, SACs, static or dynamic compression	Ca^2+^ signaling pathways, synthesis of glycosaminoglycan and matrix protein
Sánchez et al(2003)	BACs	SACs, VAHCs, membrane stretch	Na^+^ influx, H^+^ efflux, membrane depolarization
Sanchez, et al(2011)	Human articular chondrocytes	BK_Ca_ channels, membrane stretch	K^+^ efflux, membrane, hyperpolarization, matrix turnover
Sanchez et al (2010)	Articular chondrocytes from healthy human and OA patients	BK_Ca_ channels, SACs, membrane stretch	K^+^ efflux, Na^+^ influx, altered membrane potential
Hdud et al. (2014)	Equine articular chondrocytes	TRPV4, BK_Ca_ channels, membrane stretch	ERK1/2 and p38 MAPK protein phosphorylation, altered expression of TRPV4 and BK_Ca_ channels
Sanchez et al. 2011	Human articular chondrocytes	BK_Ca_ channels, membrane stretch	K^+^ efflux, membrane hyperpolarization
Lee et al. (2014)	Chondrocytes of femoral condyles from skeletally mature female pigs	Piezo1/2, cell strain in smooth lateral expanded cells under vertical compression(injurious level of strain)	Ca^2+^ influx, altered membrane potential, maladaptive chondrocyte responses, cartilage degeneration
O’Conor et al. (2014)	Articular cartilage chondrocytes from the femur and ulnas of skeletally immature pigs	TRPV4, dynamic compressive loading	Ca^2+^ influx, enhanced articular cartilage matrix synthesis and mechanical property
Zelenski et al. (2015)	Murine articular chondrocytes	TRPV4, hypotonic stress	Ca^2+^ influx and Ca^2+^ signaling pathways
Somogyi et al. (2015)	Chicken and murine articular chondrocytes	TRPV3 receptor, uniaxial cyclic compressive force	Increased expression of TRPV3 receptor, matrix synthesis
Kobayakawa et al. (2016)	HCS-2/8 cells, a human chondrocyte line	TRPV4, cyclic tensile strain	Activation of TRPV4, up-regulation of ADAM10-regulation of CD44 cleavage
Servin-Vences et al. (2017)	Primary chondrocytes from mice, HEK-293 cells	TRPV4, Piezo1, substrate-deflection, membrane stretch	Ca^2+^ influx, mechanical transduction of substrate-deflection and membrane stretch
Lv, M, et al. (2018)	Murine articular chondrocytes	TRPV4, T-type VGCCs, SACs, ECM deformation and membrane strain induced by compression	Intracellular Ca^2+^ oscillation
Yang et al. (2018)	Human articular chondrocytes from OA patients	Piezo1, cyclic stretch	Up-regulation of piezo1 protein
Karamesinis et al. (2017)	ATDC5 cell line derived from mouse teratocarcinoma cells	TRPP1,TRPP2, continuous hydrostatic pressure	Up-regulation of TRPP1 and TRPP2, regulation of chondrocyte differentiation
Du et al. (2020)	Primary chondrocytes from mice	TRPV4, piezo2, cyclic tensile strain	Ca^2+^ influx, altered membrane potential
Liu et al. (2013)	MSCs	SACs, mechanical stimulation applied on cultured MSCs by silicon nanowire	Ras/Raf/MEK/ERK signaling cascades, adhesion, chondrocyte proliferation, and differentiation of MSCs
Shimazaki, A., et al. (2006)	Human normal and OA articular chondrocytes	NMDA receptor(ligand-gated ion channels),mechanical stimuli at 0.33 Hz	Membrane hyperpolarization
Mouw, J. K et al. (2007)	BACs	Ca^2+^ channels, ATP-dependent Ca^2+^ pumps, dynamic compression	Synthesis of protein and sulfated glycosaminoglycan
Xu, B et al. (2019)	Rat articular chondrocyte	TRPV4, membrane stretch	Ca^2+^ influx
Valhmu, W. B et al. (2002)	BACs	CRACs, compressive stress of 0.1 MPa for 1 h	Compression-induced ACAN mRNA

## Mechanical responses of SACs, VGCCs and VAHCs in chondrocytes

Articular chondrocytes dwell in an environment that continuously changes their osmolarity as a consequence of mechanical stimulation. ECM deformation and hypotonic solution can generate membrane strain and result in physiological adaptation of cell volume and shape in chondrocytes. Matrix deformation-induced membrane stretching induces Ca^2+^ influx in BACs through SACs [82,83,87]. Gadolinium chloride, an SAC blocker, causes rat chondrosarcoma cells to undergo dedifferentiation accompanied by the downregulation of ACAN, SOX9, collagen 2 and collagen 9; and the upregulation of collagen 1 and fibronectin [88]. Pharmacological blockade of SACs and VGCCs can eliminate the effects of mechanical stimulation on the growth and shape development of chick joints [82]. Mechanical stimulation by silicon nanowires regulates adhesion, chondrocyte proliferation, and differentiation of MSCs partially via activation of SACs and Ras/Raf/MEK/ERK signaling cascades [68]. Cell swelling-induced membrane strain caused activation of a volume-sensitive outwardly rectifying Cl ^–^ current followed by a regulatory volume decrease in freshly dissociated rat articular chondrocytes and influenced the mechanical properties of BACs [89,90]. Membrane strains induced by hypotonic solution increase intracellular Ca^2+^ and trigger membrane depolarization in BACs via SACs [91,92]. T-type VGCCs mediate mechanically induced osteoblast-derived factor release from MC3T3-E1 cells sheared by fluid shear stress, and the conditioned-media obtained from sheared MC3T3-E1 cells may promote the early OA phenotype in chondrocytes [34]. Cyclic mechanical strain enhanced the expression of parathyroid hormone-related proteins, which promoted chondrocyte maturation and ECM formation via Ca^2+^ channels sensitive to nifedipine in chondrocytes [32]. Fluid shear stress elevated the mRNA levels of collagen Xɑ1, alkaline phosphatase (ALP), MMP13, ACAN, and collagen IIɑ1 in chondrocytes via activation of T-type VGCCs in osteoblasts and enhanced the interaction between subchondral osteoblasts and articular chondrocytes. These changes result in enhanced cartilage degeneration and subchondral bone resorption [34]. SACs and T/L-type VGCCs mediate the mechanical transduction process of immature chondrocytes in response to mechanical stretch induced by articular cartilage matrix deformation [82,93]. Mechanical stretching causes articular cartilage matrix deformation and promotes chondrocyte proliferation and chondrocyte differentiation of immature chondrocytes through SACs and L-type VGCCs and increases the mRNA and protein expression of CMP/Matrilin-1 and collagen ɑ1(X) [93].

In summary, mechanical cues are transduced, at least in part, through mechanosensitive Ca^2+^ channels, and both SACs and VGCCs participate in cartilage development and may exert synergistic effects of mechanotransduction [82]. Hypotonic solution induces membrane depolarization and intracellular alkalinization of BACs via Na^+^ influx and H^+^ efflux by SACs and VAHCs [94]. VAHCs in BACs mediate electrophysiological transduction and have specific mechanosensitive properties in response to osmotic challenges [95].

## Mechanical responses of BK_Ca_ channels in chondrocytes

Voltage-gated potassium channels play an important role in regulating the membrane potential of primary equine and elephant articular chondrocytes and may mediate the electromechanotransduction process [96]. The BK_Ca_ channel is present in the chondrocyte membrane and is highly selective for K^+^, and its gating is dependent on intracellular Ca^2+^ [27,80]. Blocking Ca^2+^-activated K^+^ channels significantly reduces the SOCE induced by Ca^2+^ addition after store-depletion of intracellular Ca^2+^ by thapsigargin in OUMS-27 cells derived from human chondrosarcoma [97]. The membrane strain induced by hypotonic solution promotes K^+^ efflux and membrane hyperpolarization by activating BK_Ca_ channels in human chondrocytes [56,80]. Hypertonic solution induced membrane hyperpolarization via K^+^ efflux by BK_Ca_ channels in human articular chondrocytes [80]. Activation of BK_Ca_ channels in human chondrocytes by hypertonic challenge may affect the synthesis and degeneration of the articular cartilage matrix via several metabolic signaling pathways [80]. The total stretch-activated membrane current is induced by the BK_Ca_ channels present in equine articular chondrocytes, as recorded by using patch-clamp electrophysiology technology [98]. TREK-2 is a mammalian two-pore domain K^+^ channel and is important for mechanosensation and can sense and transduce a broad profile of forces within the membrane [99]. Although hypertonic solution induces membrane hyperpolarization, in articular chondrocytes from healthy humans via K^+^ efflux by BK_Ca_ channels, chondrocytes from OA patients fail to respond to this kind of mechanical stimulation [100]. Stretch triggers membrane hyperpolarization via BK_Ca_ channels in isolated primary equine chondrocytes [98]. Hypertonic solutions phosphorylate p38 MAPK accompanied by altered expression of BK_Ca_ channels in equine articular chondrocytes [101].

## Mechanical responses of TRP family channels in chondrocytes

TRPV4 channels mediate significant signal transduction to osmotic and mechanical stimuli in live cells [102]. The membrane strain induced by hypotonic solution was found to activate the TRPV4 channel, and mechanically induced Ca^2+^ influx and SOX9 transcription are associated with the synthesis of articular cartilage [29,30,103]. Membrane stretching activates TRPV4 ion channels and induces Ca^2+^ influx in rat articular chondrocytes [104]. TRPV4 ion channels play a role in chondrocyte pathological processes induced by injurious levels of loading [105]. Adamalysin-like metalloproteinases with a TS motif (ADAMTS), MMPs and a disintegrin and metalloproteinase (ADAM) were reported to be expressed in cartilage and participate in the destruction of cartilage in OA by regulating ACAN expression [106,107]. Dynamic compression enhances articular cartilage matrix synthesis by upregulating the TGFβ3, collagen 2ɑ1 and ACAN genes and downregulating the ADAMTS5 gene. These effects require Ca^2+^ influx induced by mechanical activation of the TRPV4 ion channel [33,108]. The ECM of articular cartilage is a key factor in the development and progression of OA and HA plays a key role in articular cartilage lubrication and preventing ECM loss in articular cartilage in addition to its other excellent physicochemical properties, such as regulation of cell adhesion and cell motility, and manipulation of cell differentiation and proliferation [109,110]. Excessive cyclic tensile strain enhances the expression of ADAM10, which induces cleavage of CD44 a transmembrane protein that serves as an HA receptor [81]. Mechanically induced CD44 cleavage can lead to the loss of extracellular matrices in chondrocytes, which is mediated by TRPV4 in HCS-2/8 cells, a human chondrocytic cell line [81]. Treatment with the TRPV4 agonist 4ɑPDD improves the mechanical properties of articular cartilage constructs [111]. Similar to the anti-inflammatory effects in cells and explants responding to osmotic loading, cyclic tensile strain inhibited IL-1β-mediated NO and PGE2 release by activating TRPV4 channels in articular chondrocytes [112]. Isolated chondrocytes present a regulatory volume decrease, and TRPV4 plays a significant role in this process [113].

GSK1,016,790A (a selective TRPV4 agonist) promoted chondrogenesis, as evidenced by the upregulation of SOX9 and ACAN via intracellular adhesion molecule-1 [114], which demonstrated that the TRPV4 ion channel participates in cartilage growth. TRPV4 gene KO mice showed osteoarthritic knee joint degeneration and the isolated chondrocytes lost the capacity to respond to mechanical strain [29,30,103,115,116]. Chondrocytes from TRPV4 KO mice failed to respond to hypotonic stress and showed inhibition of mechanically induced Ca^2+^ influx [29,30,103]. Adipose-derived stem cells from TRPV4 knockdown mice tend to undergo adipogenic and osteogenic differentiation and resist chondrogenic differentiation, and TRPV4 knockdown mice develop severe osteoarthritis [117]. The TRPV4 agonist GSK1016790A in the absence of mechanical loading similarly enhanced anabolic and suppressed catabolic gene expression, and potently increased matrix biosynthesis and construct mechanical properties. Inhibition of TRPV4 during dynamic loading prevented acute, mechanically mediated regulation of proanabolic and anticatabolic genes, and furthermore, blocked the loading-induced enhancement of matrix accumulation and mechanical properties [33]. Pharmacological blockade and gene knockdown of TRPV4 attenuate chondrocyte apoptosis and upregulation of FAS-associated protein and cleaved caspase-3, caspase-6, caspase-7, and caspase-8 in rat OA anterior cruciate-ligament transection models [104]. Pharmaceutical activation of TRPV4 inhibited IL-1β-mediated NO release and prevented cartilage degradation and loss of mechanical properties [112]. Furthermore, activation of TRPV4 increases TRPV4 cilia translocation and modulates the expression of soluble tubulin and cilia length, suggesting the potential of TRPV4 manipulation as a novel therapeutic mechanism to suppress proinflammatory signaling and OA cartilage degradation [112]. TRPV4 contributes to the sensation of pain, which is due to hypoosmotic stimuli and inflammatory mechanical hyperalgesia, where TRPV4 sensitization by intracellular signaling leads to pain behaviors in mice [118,119]. A lack of TRPV4-mediated cartilage mechanotransduction in adulthood mice attenuates the severity of aging-associated OA. However, depletion of chondrocyte TRPV4 failed to block OA development following destabilization of the medial meniscus [116]. Impaired osmotic regulation and pressure sensation and increased knee OA scores are observed in TRPV4 KO mice [120]. Intra-articular injection of the TRPV4 antagonist RN-1734 into the knee joint appears to attenuate the responses of the sensitized C-fibers of the acutely inflamed joint to innocuous and noxious mechanical stimulation. These findings suggest that TRPV4 ion channel localization to afferent nerves may be involved in OA mechanical allodynia [121].

## Other TRP channels

Continuous hydrostatic pressure promotes runt-related transcription factor 2 production and phosphorylation of SOX9 in ATDC5 cells accompanied by the upregulation of TRPP1 and TRPP2 in ATDC5 cells [84]. Rat TRPA1 channels transiently expressed in human embryonic kidney 293 cells are activated by hypertonic solution supporting a role for TRPA1 in mechanosensation [122]. TRPA1 blockade by TCS 5,861,528 or gene depletion of TRPA1 inhibits acute inflammation, cartilage changes and joint pain by suppressing iodoacetate-induced upregulation COX-2 [123]. Upregulated expression of TRPA1 participates in the occurrence mechanism of mechanical hyperalgesia induced by OA [119]. TRPA1 is associated with the OA driving inflammatory cytokine IL-6, which is supported by high expression of IL-6, IL-6 cytokine family leukemia inhibitory factor and IL-11 in WT chondrocytes and significant downregulation of IL-6 in TRPA1 KO mice. Furthermore, treatment with a TRPA1 antagonist significantly downregulated the expression of IL-6 in chondrocytes from WT mice and OA patients, which indicates that TRPA1 regulates the synthesis of the OA driven inflammatory cytokine IL-6 in chondrocytes and may serve as a possible therapeutic target for OA [124]. TRPV3 receptors mediate the mechanical transduction process and the uniaxial cyclic compressive force increases the expression of the TRPV3 receptor in chondrocytes [78]. The mRNA expression of TRPV3 is the highest among TRPV family channels in chicken and mouse tissue samples [78]. Although the mRNA expression of TRPV3 is significantly induced by mechanical load as revealed by semiquantitative RT-PCR analyses, the mRNA expression of other TRPV channels fails to show any significant alterations upon mechanical load [78]. The resting membrane potential of chondrocytes may be partially controlled by TRPV5 and the positive resting membrane potential may play a protective role in chondrocytes responding to hypotonic solution with minimum changes in cell volume [71,125].

## Mechanical responses of Piezo1/2 channels in chondrocytes

Piezo is a type of mechanically sensitive ion channel that is necessary for cells to respond to mechanical stimuli and can convert mechanical signals sensed by the membrane into intracellular electrical or chemical signals. Crosslinking between the cap and blade residues inhibits mechanical gating of the Piezo1 channel [126]. Cyclic stretch increased the expression of Piezo1 channels in human articular chondrocytes from OA patients [127]. Cholesterol enrichment or depletion by methyl-β-cyclodextrin or disruption of membrane cholesterol organization by dynasore can impair the function of Piezo1 channels. These findings highlight the importance of plasma membrane localization and organization of Piezo1 channels and indicate that mechanical activation of the Piezo1 channel is directly dependent on the membrane composition and lateral organization of membrane cholesterol domains [128]. Similar to mechanically induced Piezo1 activation, Piezo1 activation is countered by the K^+^ channel TRAAK (K2P4.1). This action is also responsive to mechanical forces [129]. This finding suggests that although membrane tension is the direct mediator of force, other mechanosensitive ion channels can actually play important indirect roles by influencing which regions of the cell membrane experience changes in tension when forces are applied to a cell. Interestingly, mechanical stimulation of the lipid bilayer alone is sufficient to activate Piezo1 channels, which indicates that Piezo1 channels functioning as molecular force transducers in the cellular membrane may not rely on alternate cellular components to sense mechanical stimuli [130,131]. Electrophysiology experiments of Piezo1/2 channels confirm that Piezo1/2 channels function as significant frequency filters. The effectiveness of mechanical transduction varies with the stimulus frequency, waveform, and duration presented by patch-clamp recordings, which suggests the potential contributions of Piezo1/2 channels in transducing repetitive mechanical stimuli [132]. In addition to transduction of extracellular mechanical forces, Piezo1 channels may be activated by cell-generated forces induced by Myosin II phosphorylation by Myosin Light Chain Kinase [133]. Application of a negative pressure failed to activate Piezo2 channels in human Merkel cell carcinoma cells and human embryonic kidney 293 T cells, but both positive and negative pressure activated Piezo1 channels in a similar manner [134]. We also note that moderate cold can potentiate the conversion of mechanical force into excitatory current because of the cold sensitivity of Piezo2 channels and Piezo2 channels are evolutionarily conserved as cutaneous mechanoreceptors [135], which may provide evidence for the involvement of Piezo2 channels in chondrocyte mechanotransduction process. TRPV4 and Piezo1 channels mediate compression-induced chondrocyte membrane strain and result in intracellular Ca^2+^ concentration oscillation in the cytoplasm [35,86]. The physiological level of intermittent tensile strain caused TRPV4-dependent membrane potential, but injurious levels of intermittent tensile strain triggered Piezo2-dependent membrane potential. In conclusion, these findings thus reveal the key role of TRPV4 and Piezo2 ion channels in mechanoelectrical transduction in primary murine chondrocytes [85].

Piezo1 and Piezo2 gene KO mice presented articular cartilage defects, which indicated that Piezo1 and Piezo2 ion channels are vital to articular cartilage growth and regulate the mechanical properties of the ECM and embedded chondrocytes [136]. GsMTx4 (Piezo1/2 channel inhibitor) attenuated the deteriorated response of chondrocytes to injurious mechanical strain [105]. GsMTx4 and Piezo1/2 siRNA attenuated injurious levels of strain-induced maladaptive murine chondrocyte responses, which demonstrated that piezo1/2 participates in cartilage injury and posttraumatic OA [105,137]. GsMTx4 may play a chondroprotective role in mechanically induced articular cartilage deterioration by joint disability [33,105]. The endogenous peptide urocortin1 can maintain Piezo1 channels in a closed conformation and firmly protect articular cartilage and chondrocytes against corticotropin-releasing factor receptor 1 selective antagonist CP-154,526–induced accumulation of intracellular Ca^2+^, which is due to opening nonselective cation channels and cell death [138].

## Mechanical responses of P2 receptors and CRACs in chondrocytes

Membrane depolarization in OA chondrocytes and membrane hyperpolarization in normal chondrocytes suggested that the purinergic signaling pathways are important mediators in OA progression [139]. Mechanically induced ATP release and an increase in extracellular ATP levels activate ligand-gated ion channel P2X receptors and some G-protein-coupled P2Y receptors, and these effects can regulate chondrocyte function and modulate intercellular communication [140]. Dynamic compression-induced ATP release in chondrocytes activates P2 receptor ion channels. For instance, the P2X7 receptors and subsequent purinergic signaling pathways, including the Ca^2+^ influx and Ca^2+^ signaling pathways, are activated, which then stimulate the synthesis and release of proteoglycans in chondrocytes and prevent compression-induced NO release of chondrocytes and articular cartilage degeneration [24,141]. Unlike other ion channels that are stimulated or activated directly by mechanical forces, P2X and P2Y receptor ion channels are both activated indirectly by a mechanically induced release of nucleotides such as ATP. Mechanically induced ATP release activates P2Y2 receptors and alters membrane potential in human chondrocytes [139]. Purinergic receptors can also confer the mechanosensitivity of the voltage-gated K^+^ channel KCNQ1 (also known as Kv7.1 or KvLQT1). Static compression induced the expression of ACAN mRNA in BACs, which can be dose-dependently or completely blocked by antagonists of the Ca^2+^/CaM signaling pathway and thapsigargin that deplete IP_3_-sensitive intracellular Ca^2+^ stores [53]. Both the amplitude and the maximal rate of rise of SOCE are significantly reduced by SOCE blockers, which decrease the mRNA expression of ACAN and collagen 2 decreases severely in chicken chondrogenic mesenchymal cells [73], which may provide substantial evidence that intracellular Ca^2+^ stores are vital for Ca^2+^ oscillations and chondrogenesis. Orai1, Orai2 and STIM1 form functional CRACs in OUMS-2 cells, and these complexes are responsible for sustained Ca^2+^ entry in response to histamine stimulation [74]. Histamine-induced SOCE through CRACs may also contribute to mechanically induced intracellular Ca^2+^ increases in chondrocytes.

## Mechanical responses of NMDA receptors, ASICs, chloride channels and DEG/ENaCs in chondrocytes

One of the ionotropic glutamate receptors, the NMDA receptor, is expressed in human articular chondrocytes. This receptor may mediate mechanically induced membrane hyperpolarization [142]. Ca^2+^/calmodulin-dependent protein kinase II (CaMKII), which is associated with NMDA signaling, has four subunit isoforms (alpha, beta, gamma, delta). CaMKII may mediate a mechanically induced increase in intracellular Ca^2+^ in a wide variety of cells and tissues. The alpha – and beta-isoforms have narrow distributions restricted mainly to neuronal tissues, but the gamma – and delta-isoforms are ubiquitously expressed within neuronal tissue and articular cartilage. The CaMKII isoforms gamma and delta are expressed in both normal and OA chondrocytes and are only involved in the response of normal chondrocytes to mechanical stimulation [142]. ASIC3 is present in murine articular chondrocytes and participates in OA progression, and murine ASIC1b is permeable to K^+^ and functions as a mechanogating ion channel responding to membrane stretching [143]. Hypotonic stimuli significantly enhance acid-evoked membrane currents via ASIC1b in oocytes [143]. Cl ^–^ channels are known to be expressed in mammalian chondrocytes, such as voltage-dependent Cl ^–^ channels and swelling-activated Cl ^–^ channels, and these Cl ^–^ channels can participate in the regulation of resting membrane potential, cell volume, cell survival, and endochondral bone formation [144]. Chloride channels regulate chondrogenesis in chicken mandibular mesenchymal cells [145]. Hypotonic solution and extracellular acidification elicited a volume-sensitive outwardly rectifying Cl ^–^ current followed by a regulatory volume decrease and an acid-sensitive outwardly rectifying Cl ^–^ current, respectively [146]. Hypotonic solution-induced Cl ^–^ current and regulatory volume decrease responses occurred in isolated rabbit and articular chondrocytes [89,147,148]. The CIC-3 chloride channel is responsible for hypotonic solution-induced Cl – current and regulatory volume decrease in response to hypoosmotic environments [149]. Anterior cruciate ligament transection treatment results in a large increase in hypotonic-activated chloride conductance in rabbit chondrocytes and enhanced caspase-3/7 activity followed by the onset of apparent cartilage loss [150]. Hypotonic solution decreases the expression level of the ClC-7 chloride channel, which may participate in OA progression in human chondrocytes [151]. Tyrosine phosphorylation induced by the protein tyrosine kinase regulates the functional role of hypotonic solution-induced Cl ^–^ current in the regulatory volume decrease of isolated rabbit articular chondrocytes [152]. The DEG/ENaC (degenerin/epithelial sodium channel) protein family comprises related ion channel subunits from all metazoans, including humans. Members of this protein family play roles in mechanotransduction. DEG/ENaC-like ion channels participate in the modulation of canine chondrocyte volume, which can be inhibited by benzamil by reducing the influx of Na^+^ ions [153]. The DEG/ENaC ion channel complex may contribute to mechanically induced chondrocyte dysfunction and OA pathological changes [154].

## Articular joint response to hind limb unloading or simulated microgravity

In addition to different mechanical stimuli, decreased mechanical forces or mechanical unloading situations simulated by the hind limb, joint immobilization and the random positioning system (RPM) also regulated the functions of articular chondrocytes and were accompanied by altered expression of mechanosensitive ion channels. Mechanically induced lubricant HA secretion by synoviocytes can maintain articular cartilage [155,156], which may contribute to the effects of mechanical loading of articular cartilage on the metabolism of resident chondrocytes and the synthesis of molecules to maintain the integrity of the cartilage. Moderate exercise promotes proteoglycan synthesis and maintains articular ECM homeostasis. However, articular joint immobilization may reduce HA secretion and result in decreased cartilage thickness and inhibited proteoglycan synthesis leading to ECM degeneration, which will make the articular cartilage more vulnerable to excessive mechanical stimulation. The chondrocyte phenotype was preserved when suspended clustered chondrocytes maintained a round morphology under simulated microgravity induced by RPM [157]. Progressive dedifferentiation which includes the production proportion of collagen 2/collagen 1, proteoglycan proportion of ACNA to versican (ACNA/VCAN) and a transition to fibroblast-like morphology was observed in monolayer in vitro cultured BACs except for decreased expression of the TRPV4 ion channel, which was attenuated by the RPM system that induced the suspension status of in vitro BACs [157]. Rats subjected to mechanical unloading induced by the hind limb presented increased expression of inducible NO synthase, enhanced chondrocyte apoptosis, decreased thickness of articular cartilage and compromised joint biomechanics [33]. Male C57BL/6 J mice under mechanical unloading conditions such as hind limb and joint immobilization presented with subchondral bone atrophy accompanied by osteoclast differentiation of bone marrow cells and degeneration of the cartilaginous layer without chondrocyte hypertrophy [158]. In addition, increased ALP and aggercanase activity, decreased ACAN content in calcified cartilage and decreased ALP activity in calcified cartilage were observed in male C57BL/6 J mice in the hind limb and joint immobilization groups [158]. As shown in Table 2, the expression of mechanosensitive TRPV4 channels present in articular chondrocytes is changed during progressive osteoarthritis cartilage degeneration, which is consistent with these important roles of mechanosensitive ion channels in OA progression.

**Table 2.** Articular chondrocytes in response to hind limb unloading or simulated microgravity

Study	Cell/Tissue	Decreased mechanical stimulation	Results
Wuest et al. (2018)	BACs	TRPV4, simulated microgravity induced by RPM	Altered expression of TRPV4, preserved chondrocyte phenotype
Basso et al(2006)	Articular and growth plate cartilage	Hind limb	Increased expression of iNOS, impaired articular cartilage, deteriorated joint biomechanics
Nomura et al. (2017)	Male C57BL/6 J mice	Hind limb, joint immobilization	Altered ALP and aggercanase activity, decreased ACAN content subchondral bone atrophy, cartilage degeneration

## Chondrocytes in OA

Chondrocytes are the cells within cartilage that produce and maintain the extracellular matrix. Volume regulation of chondrocytes is vital to their function and occurs in OA [113]. Articular chondrocytes are exposed to changing osmolarity and compressive loads. Ion channels implicated in volume control are changed in chondrocytes from osteoarthritic cartilage [113]. In addition to mechanically induced chondrocyte phenotype changes, some specific mechanosensitive ion channel agonists or antagonists and gene knockdown technology can also alter the chondrocyte phenotype and may be involved in OA pathological progression and OA pain. As shown in Table 3, some mutations in TRPV4 cause severe developmental abnormalities, such as skeletal dysplasia and arthropathy [118]. Differential regulation of ion channels such as BK_Ca_ and TRPV4 at the functional level and expression level is observed in early OA synovial fluid mesenchymal progenitor cells [159,160], which suggests that mechanosensitive ion channels participate in OA progression.

**Table 3.** The role of ion channels in osteoarthritis

Study	Cell/Tissue	Ion channel and intenvention	Results
Funabashi et al. (2010)	OUMS-27 cell line	SK channels, CRAC channels, histamine	K^+^ efflux through SK channels, increased intracellular Ca^2+^ concentration via nonselective cation channels including CRAC channels, membrane hyperpolarization
Li et al. (2017)	Human chondrocytes from OA patients	Piezo1, GsMTx4, a PIEZO-blocking peptide	Suppressed expression of apoptosis-related genes
Sooampon et al. (2013)	Human periodontal ligament (HPDL) cells, human osteoblasts	Piezo1, GsMTx4	Ca^2+^ influx, attenuated deteriorated response of chondrocytes to injurious mechanical strain
O’Conor et al. (2016)	Conditional knockout (cKO) mice	Gene knock down of TRPV4 channel	Decreased total periarticular bone volume, reduced severity of aging-associated OA
Ogawa et al. (2019)	The ATDC5 cell line	TRPV4, GSK1016790A, a selective TRPV4 agonist	Activation of TRPV4-ICAM-1-up-regulation of chondrogenic marker genes including *SOX9* and *ACAN*, HA facilitated TRPV4-induced chondrogenesis
Srinivasan et al. (2015)	KO and WT mice	Gene knock down of T-type VGCCs	Enhanced cartilage degeneration and subchondral bone resorption
Kuduk, S. D., et al. (2010), Izumi, M., et al. (2012)	KO and WT mice, rat osteoarthritis models	Gene knock down of ASIC-3, the ASIC3 inhibitor A-317,567, ASIC3 selective peptide blocker (APETx2)	Altered expression of ASIC3 in knee joint afferents, reversed osteoarthritis pain and mechanical hyperalgesia
Schuelert, N., et al. (2010)	Rat osteoarthritis models	The cannabinoid CB2 receptor agonist GW405833	Reduced mechanosensitivity of afferent nerve fibers in control joints, nociceptive responses in OA joints
Shimazaki, A., et al. (2006)	Human normal and OA articular chondrocytes	NMDA receptor(ligand-gated ion channels), CaMKII inhibitor	Inhibited membrane potential, upregulation of aggrecan mRNA
Clark et al. (2010)	KO mice	Gene knock down of TRPV4	Severe OA degeneration
Lee et al. (2014)	Murine chondrocytes	Piezo1, GsMTx4, siRNA	Attenuated maladaptive chondrocyte responses
Zelenski et al. (2015)	Murine articular chondrocytes	Gene knock down of TRPV4	Suppressed mechanically induced Ca^2+^ influx and formation of pericellular matrix
Moilanen, L. J., et al. (2015)	Murine articular chondrocytes	Pharmacological blockade and gene knock down of TRPA1	Suppressed iodoacetate-induced OA
O’Conor, C. J., et al. (2016)	cKO mice	Gene knock down of TRPV4	Reduced severity of aging-associated OA
He, B. H., et al. (2017)	OA mice	Mechanosensitive ion channels, the selective inhibitor GsMTx4	Reduced activation of dorsal horn nociceptive circuits and primary mechanical allodynia
Xing, R., et al. (2017)	OA mice	TRPA1, TRPV4	Upregulation of TRPA1 and TRPV4 mechanical hyperalgesia induced by OA
Parisi, C., et al. (2018)	Chick joint	Pharmacological blockade of SACs, VGCCs,	Removed effects of mechanical stimulation on joint cartilage growth and shape development
Raouf, R., et al. (2018)	Rat DRG nerons	Piezo2, overexpression and gene knock down of annexin A6	Attenuated mechanical hyperalgesis induced by OA
Richter, F., et al. (2019)	Rat DRG nerons	TRPV4, agonist 4αPDD, GSK 1016790 A and antagonist RN-1734	Altered mechanonociception of the normal and inflamed joint
Xu, B., et al. (2019)	Rat articular chondrocytes	Pharmacological blockade and gene knock down of TRPV4	Attenuated cartilage degeneration in rat OA anterior cruciate-ligament transection (ALCT) models
Nummenmaa, E., et al. (2020)	Rat articular chondrocytes, human OA chondrocytes	Pharmacological blockade and gene knock down of TRPA1	Downregulation of the pro-inflammatory cytokine interleukin-6 (IL-6), IL-6 family cytokines leukemia inhibitory factor (LIF) and IL-11
Fu, S., et al. (2021)	Rat articular chondrocytes	Pharmacological blockade or activation of TRPV4	Pro-inflammatory signaling and cartilage degradation

GsMTx4 inhibits the activation of nociceptors followed by mechanical allodynia by weakening the sensitization of mechanosensitive ion channels in OA mice [161], which indicates that Piezo1/2 channels also participate in OA mechanical hyperalgesia in addition to OA cartilage degeneration. Annexin A6 is a membrane-associated Ca^2+^-binding protein. Annexin A6-deficient mice showed increased sensitivity to mechanical stimuli and increased activity of Piezo2 channels that mediate a rapidly adapting mechanogated current linked to proprioception and touch in sensory neurons. Overexpression of Annexin A6 may attenuate OA mechanical pain by inhibiting rapidly adapting currents induced by Piezo2 channels [162]. Intra-articular injection of VGCC inhibition may lessen OA changes, such as enhanced cartilage degeneration and subchondral bone resorption, in both posttraumatic and age-related OA [34]. Dysregulation of NMDA-CaMKII signaling may contribute to the onset and progression of osteoarthritis [142]. Upregulation of pain-related neurochemical markers such as ASIC3 is observed in joint afferents of a rat OA model induced by intra-articular injection of monoiodoacetate [163]. The ASIC3 inhibitor attenuated mechanical hyperalgesia by inhibiting the expression of ASIC3 in knee joint afferents and ASIC3 KO mice reversed mechanical hyperalgesia in a rat osteoarthritis model, which indicates the involvement of ASICs in osteoarthritis pain via the central nervous system [164,165]. The intracellular injection of the CB2 receptor agonist GW405833 into synoviocytes attenuates osteoarthritis knee joint pain in a rat osteoarthritis model [166].

In summary, mechanical stimulus mechanically induced intracellular cation mobilization via ion channels, such as TRPV4, ASIC3 and Piezo1/2, and these mechanosensitive channels participate in mechanically induced alteration of articular chondrocytes and the maintenance of ECM homeostasis in joints. Ion channels may serve as potential biomarkers for OA [167]. The intensification of ion channels may prevent or attenuate osteoarthritis progression. Dysregulation of the mechanical responses of chondrocytes may participate in the progression of OA [100].

## Conclusion and perspectives

During joint movement, the mechanical load of body weight is the most physiological stimulus affecting cartilage. Body weight can influence of the ECM in applied animal experiments. Excessive obesity may induce injurious levels of mechanical stimulation and thus accelerate joint aging. However, significantly decreased mechanical stimuli, such as hind limb unloading, can also cause pathological degeneration of articular cartilage because of a lack of sufficient physiological mechanical stimulation. Moderate exercise under healthy body weight may exert chondroprotective effects and stimulate the anabolic metabolism of articular cartilage in physiological mechanical contexts. Obesity or fracture of the articular surface can result in a quantitative imbalance between anabolic and catabolic activity, along with ECM catabolic metabolism, particularly in swelling articular cartilage tissue, which leads to the development of OA and compromised mechanical resilience of articular cartilage. As a result, it is important to investigate the effects of deformation pressure or shear force on articular cartilage homeostasis. These data can unveil the pathophysiological properties of OA progression by exploring the molecular mechanism by which mechanoreceptors participate in the mechano-adaptation process.

In many different cell biomechanical studies, there is a direct link between mechanoreceptors at the cell surface and intercellular biochemical signaling, which in turn controls downstream effector molecules [168]. Among the mechanoreceptors in the cell membrane, mechanosensitive ion channels are essential for the transduction of mechanical stimuli into biologically relevant signals. The innate force-sensing ability of mechanosensitive channels transduces mechanical signals. Furthermore, mechanical stimuli can induce alteration in chondrocyte metabolism and influence the homeostasis of articular cartilage. Although physiological expression of ion channels is essential for articular ECM formation, differentially expressed transmembrane channels on chondrocyte surface membranes (plasmalemma) are relevant to OA [27,83,141]. The effects of mechanosensitive ion channels may delay OA cartilage degeneration and attenuate OA-induced mechanical allodynia. The molecular mechanisms by which ion channels participate in the mechanotransduction process are not clear, and the interaction relationships among some of these ion channels are unknown. More research is needed to define the electrophysiological properties of individual ion channels that can participate in mechanically altered metabolism of chondrocytes. The molecular mechanisms by which mechanical stimuli prevent articular cartilage degeneration and promote articular cartilage repair may yield insights into the rationale of possible mechanical therapy, such as the potential for therapeutic low-intensity pulsed ultrasound for OA [169,170].
